# Environmentally sensitive grain-size component records and its response to climatic and anthropogenic influences in Bosten Lake region, China

**DOI:** 10.1038/s41598-020-57921-y

**Published:** 2020-01-22

**Authors:** Long Ma, Jilili Abuduwaili, Wen Liu

**Affiliations:** 1 State Key Laboratory of Desert and Oasis Ecology, Xinjiang Institute of Ecology and Geography, Chinese Academy of Sciences, Urumqi, 830011 China; 2 Research Center for Ecology and Environment of Central Asia, Chinese Academy of Sciences, Urumqi, 830011 China; 3 University of Chinese Academy of Sciences, Beijing, 10049 China

**Keywords:** Limnology, Sedimentology

## Abstract

Using ^137^Cs and ^210^Pb dating and multi-proxy evidence from a 41-cm sediment core from Bosten Lake in China, the responses of sediment grain size to environmental changes were reconstructed over the past 150 years. After the end of the Little Ice Age, the climate of the Bosten Lake region became warmer and drier, and the lake water level decreased. The results indicated that the lowest water storage periods occurred at approximately 1920–1930 AD. Decreases in the Siberian High intensity and water vapour transport from the Indian Ocean during this period led to a reduction in the water vapour supply, which resulted in reduced lake levels in the period 1920–1930 AD. Then, the lake was at a high level until the 1960s. The water storage then declined in the 1960s. Since the 1960s, the contents of total organic carbon and total nitrogen have significantly decreased, which is closely related to the significant decline in water level and increased water salinity caused by enhanced water demands. Increased irrigation water demand as a result of expanding cultivated areas and climate change, coupled with a reduced input of water vapour, resulted in the worst water environment in approximately 1980–1990 AD. Since the late 1980s, the water level of the lake has risen, and the lake primary productivity of Bosten Lake has improved. Through the application of statistical methods to grain size data from Bosten Lake combined with the abovementioned data on climate change and human activities, two major potential factors influencing the grain size of terrigenous clastic material were revealed. The first factor, consistent with a grain size of 3.31 μm, is related to the recent increase in agricultural acreage in the Bosten Lake watershed and may reflect increases in atmospheric dust. The second factor, correlated with grain sizes of 11.48 μm and 69.18 μm, can be used to reflect changes in the lake hydrological state. It is suggested that the grain sizes of these lake sediments sensitively reflect changes in the hydrological characteristics of the basin and can be used to reconstruct the history of climate change and human activities.

## Introduction

Lake sediments record the history of human-environment interactions^1^ and have been used to reconstruct the environmental evolution on different time scales and to assess the impacts of human activities^2^. Among the multiple proxies derived from lake sediments, the grain-size distribution has been widely used to reconstruct environmental histories^3–7^. Variations in sedimentary environments are reflected by polymodal grain-size distributions, which represent changes in transport and depositional processes^8,9^. Weibull^8^ and unimodal lognormal distributions^10^, end-member mixing analysis^11,12^, and standard deviation variations^13^ have been used to obtain environmental grain-size compositions and to distinguish sediment sources.

Lakes in arid regions play significant roles in maintaining regional ecosystems. Deteriorations in water quality and quantity can threaten the livelihoods of local people and the biological diversity of lake wetlands. Under the combined influence of human activities and climate change, rapid changes in lakes in the arid regions of Central Asia, such as the Aral Sea^14^, Bosten Lake^15^, Ebinur Lake^16^, and Lop Nur^17^, have raised concerns in the past few decades. Bosten Lake lies in arid Central Asia and is ideally located for studying the environmental changes in regions with climates dominated by westerlies^18^. For Bosten Lake, modern lake environmental changes, including changes in water quality and quantity^19–23^ and changes in surface lake sediments^24–28^, are increasingly the focus of research, and some studies have also discussed palaeo-environmental changes over long time scales^18,26,29–32^. Organic carbon in surface sediments^33^ and carbon burial over the past century have also been used to reveal the important role of Bosten Lake in the terrestrial carbon cycle^34^. Despite the existing body of research of Bosten Lake, few studies have focused on the sediment grain-size distribution in response to changes in the lake environment in response to climatic and anthropogenic influences, which could provide insights into the underlying mechanisms forcing environmental changes under the superimposed influences of climate change and intensified human activity.

This paper aims to link the grain-size composition from lake sediment to the lake level status of Bosten Lake through statistical methods combined with instrumental data. The results will enrich the palaeo-environmental significance of multiple proxies from lake sediments and will be used to better understand the important role of human activities in influencing lake environmental change.

## Geographical Setting

**Figure 1:**
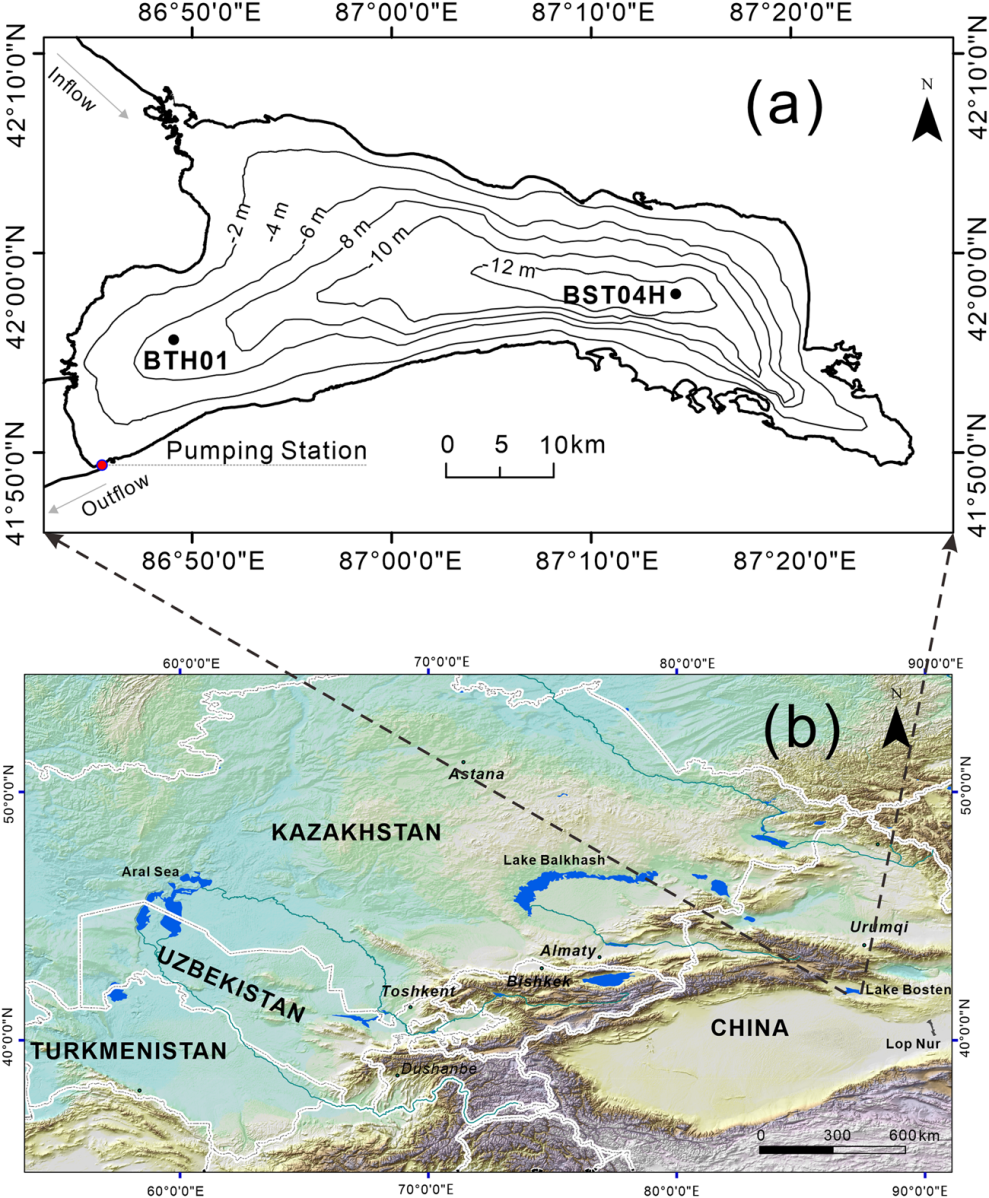
Locations of Bosten Lake and sample cores BTH01 and BST04H^36^ for comparison with the time horizon based on ^137^CS. (**a**) Bathymetric map of Bosten Lake and the core location and (**b**) location of Bosten Lake in Central Asia. The source of the base maps is the NASA Shuttle Radar Topographic Mission 90 m Digital Elevation Data^35^.

The Bosten Lake basin lies between the Tian Shan Mountains and the Taklamakan Desert (Fig. 1, digital elevation data from the CGIAR-CSI SRTM 90 m Database^35^) and has a typical arid climate^36^. The total annual precipitation is only 76.1 mm; however, evaporation amounts to 2000 mm year^−1^ ^18^. The Bosten Lake catchment comprises four counties, namely, Yanqi, Hejing, Heshuo, and Bohu, which have experienced rapid economic development over the past half century. Bosten Lake was once the largest inland freshwater lake in China, with a surface area of 1005 km^2^ and a catchment area of 4.5 × 10^4^ km^2^,^37^. The lake receives water from several rivers, including the Kaidu, Huang, and Qing Rivers, but only the Kaidu River is perennial. The Kaidu River originates in the snow-capped central part of the Tian Shan Mountains and is supplied by melted snow and ice. It has a total length of 513 km, a drainage area of 2.2 × 10^4^ km^2^, and an annual runoff of 34.1 × 10^8^ m^3^.

Bosten Lake has undergone significant changes due to human activities; for example, water levels have changed sharply with fluctuations ranging from 1045 to 1049 m^38^. The salinity showed an increasing trend, with values of 0.4 g/L in 1958–1960, 1.60–1.87 g/L in 1980–1991, 1.17 g/L after 1992, and 1.48 g/L in 2008^37,39^.

## Results

### Variations in multiple environmental proxies from Bosten lake sediments

**Figure 2:**
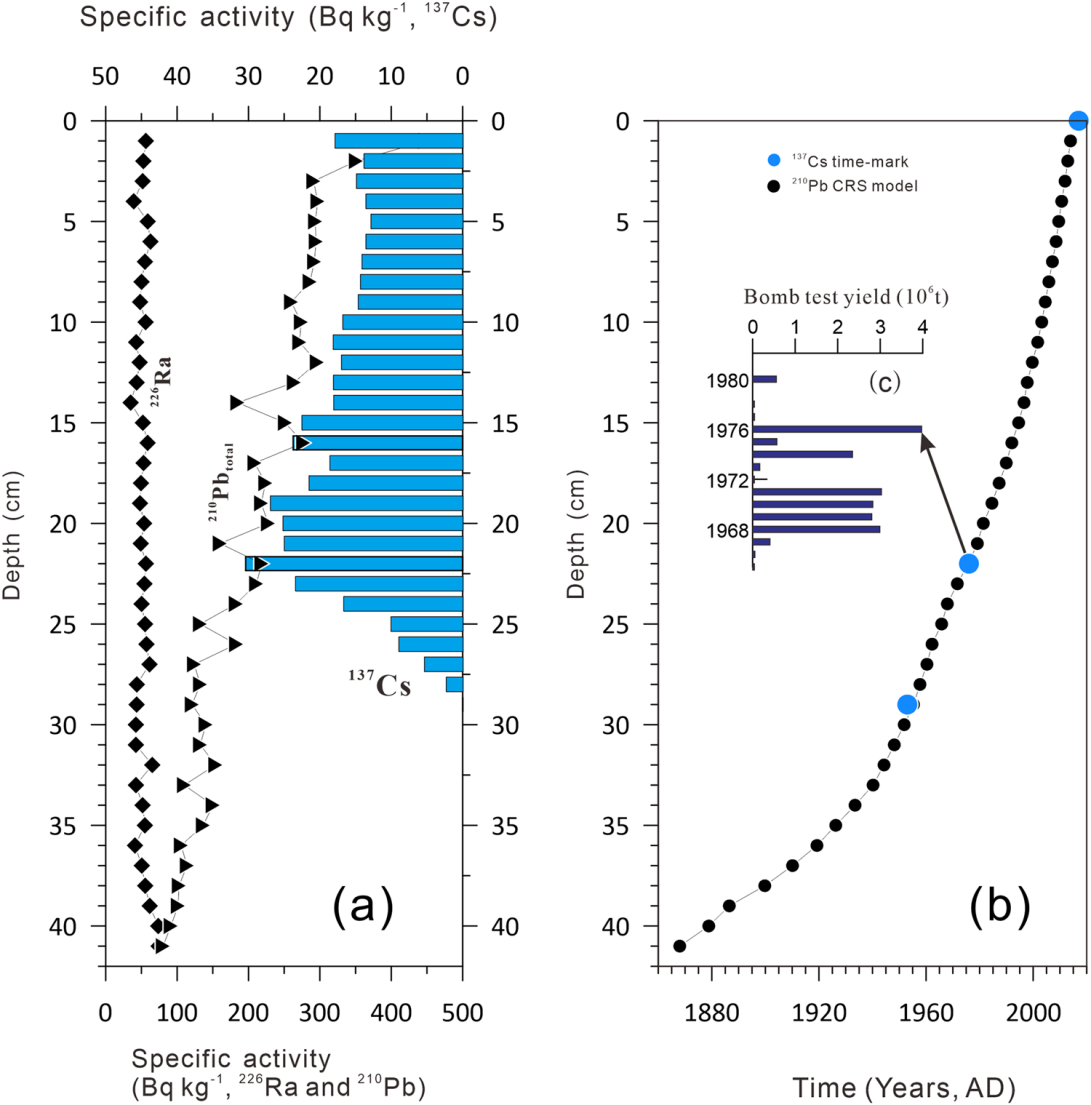
Chronosequence of the Bosten Lake core BTH01. (**a**) The specific activities of ^210^Pb and ^226^Ra in core BTH01. (**b**) Age-depth model of sediment core BTH01. (**c**) The yields of Chinese bomb tests^36^.

The specific activity of excess ^210^Pb was determined by measuring the amount of ^210^Pb and ^226^ Ra in the same layer. ^210^Pb and ^226^Ra were equilibrated at 39 cm, and the chronology of the sediment was established by the constant rate of supply (CRS) model with supported ^210^Pb_ex_ (^210^Pb_ex_ = ^210^Pb-^226^Ra)^40^. The specific activity of ^137^Cs is shown in Fig. 2a. The global fallout of ^137^Cs in 1954 AD^41^ has been identified in the Lake Bosten sediment core at 29 cm. In general, the ^137^Cs fallout peak should correspond to 1963 AD; however, the Lake Bosten watershed was a unique nuclear test zone in China. The core sediments recorded local bomb tests in 1976 AD^36^ (the time horizon of 1976 AD was also recorded in BST04H^36^ in Fig. 1) (Fig. 2c), and the results are in good agreement with the ^210^Pb dating model^42^ (Fig. 2b).

**Figure 3:**
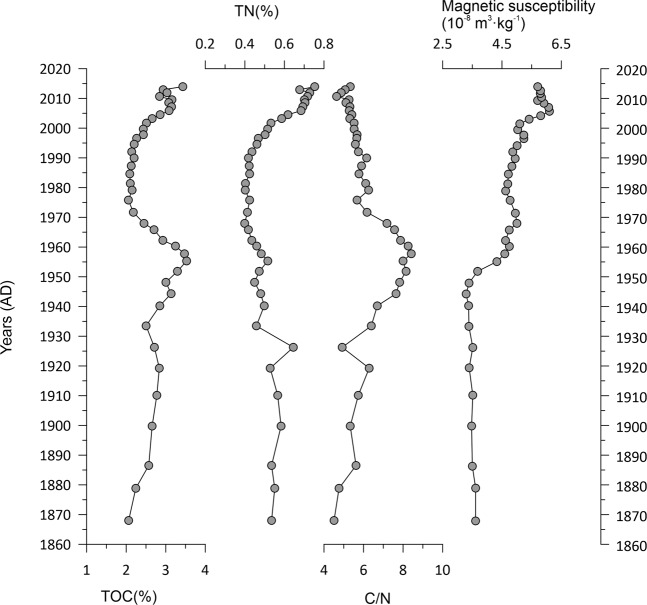
Sedimentary profiles of organic matter indices (TOC, TN, and C/N) and magnetic susceptibility (MS) for the Lake Bosten sediment core BTH01.

**Figure 4:**
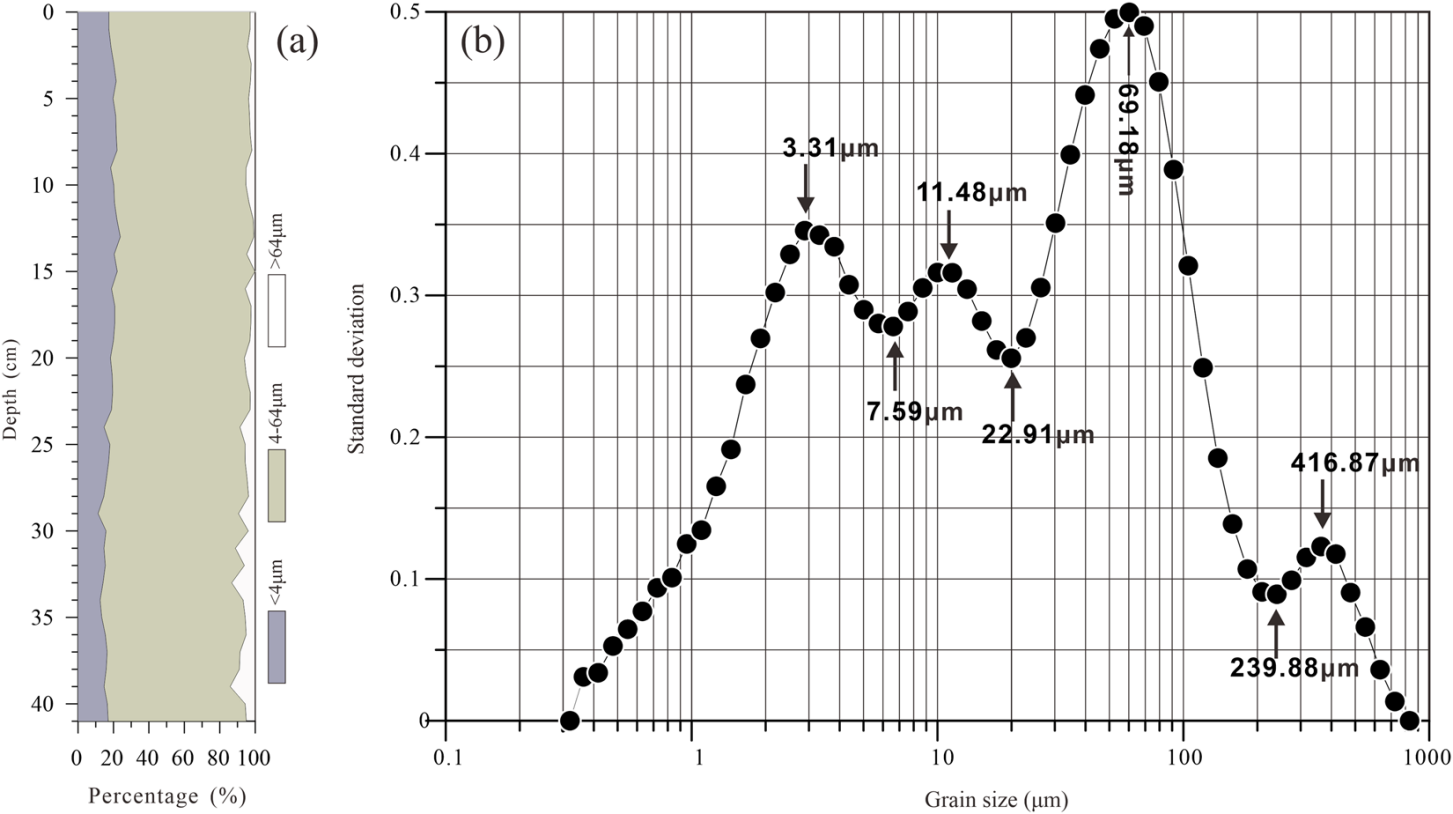
Grain-size characteristics of Bosten Lake sediments. (**a**) Grain-size distribution of core sediment BTH01. (**b**) Standard deviation values versus grain size of sediment core BTH01.

The variations in the organic geochemical proxies in the core sediments from Lake Bosten are shown in Fig. 3. The average value of magnetic susceptibility (MS) is 4.7 × 10^−8^ m^3^·kg^−1^. Before 1955 AD, the MS is low, with an average of 3.5 × 10^−8^ m^3^·kg^−1^. Since 1955 AD, the MS has increased obviously, with an average of 5.2 × 10^−8^ m^3^·kg^−1^. The average content of total organic carbon (TOC) is 2.7%, with a maximum of 3.5% and a minimum of 2.1%. From the base to 1960 AD, there is an increasing trend in the TOC content. Then, the TOC content decreases until 1980 AD, with a minimum value of 2.1%. Since 1980 AD, the TOC content has gradually increased again. The average content of total nitrogen (TN) in the sediments is 0.53%, with a maximum of 0.75% and a minimum value of 0.40%. There are obvious similarities between the TOC and TN contents. The average C/N ratio in the sediments of Bosten Lake is 6.1. The maximum value is 8.4 in ca. 1960 AD. For Bosten Lake sediments, the different grain size fractions of >64 μm, 4–64 μm and < 4 μm accounted for 5%, 76.9% and 18% of the sediment, respectively (Fig. 4).

### Environmentally sensitive grain-size components extracted via the grain-size standard deviation method and factor analysis

**Figure 5:**
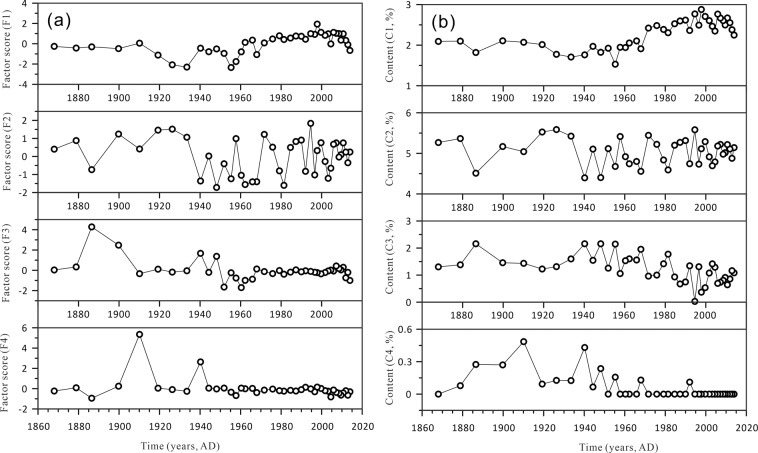
(**a**) The four factor scores retained from factor analysis (F1, F2, F3 and F4) and (**b**) the contents of the four peaks, C1 (3.31 μm), C2 (11.48 μm), C3 (69.18 μm) and C4 (416.87 μm), extracted from the curve of the standard deviation versus grain size.

Based on extraction via the grain size-standard deviation method (Fig. 4), there are four peaks in the grain-size standard deviation curve, which are sensitively influenced by the sedimentary environments. The three boundaries for the sub-populations of Bosten Lake sediments were approximately 7.59 μm, 22.91 μm, and 239.88 μm. Sub-population S1 include grain sizes from 0 to 7.59 μm and has contents of 26.13% to 44.88%. Sub-population S2 has contents ranging from 34.90% to 43.03% and includes grain sizes from 7.59 to 22.91 μm. Sub-population S3 has contents ranging from 14.38% to 34.56% and includes the grain sizes from 22.91 to 239.88 μm. Sub-population S4 has contents of less than 2.65% and includes grain sizes greater than 239.88 μm. The contents of the four peaks, C1 (3.31 μm), C2 (11.48 μm), C3 (69.18 μm) and C4 (416.87 μm), are displayed versus time in Fig. 5.

Factor analysis is generally used to determine source apportionments and to identify environmental influencing factors in limnological studies^43–45^. As shown in Table 1, four factors explain 94.6% of the total variance. The factor scores of the first four factors are shown in Fig. 5. The four factors (F1, F2, F3 and F4) account for 43.6%, 23.2%, 18.7%, and 9.2% of the total variance, respectively. The factor score distributions for the abovementioned four factors are reported in Fig. 5.

**Table 1:** Total variance and component matrixes (four selected factors) for the grain size data from the Bosten Lake sediment core BTH01.

Component^a^	Initial Eigenvalue			Extraction Sums of Squared Loadings			Rotation Sums of Squared Loadings		
	Total	% of Variance	Cumulative %	Total	% of Variance	Cumulative %	Total	% of Variance	Cumulative %
1	32.685	59.426	59.426	32.685	59.426	59.426	23.982	43.604	43.604
2	10.567	19.213	78.640	10.567	19.213	78.640	12.749	23.181	66.785
3	6.563	11.932	90.572	6.563	11.932	90.572	10.267	18.666	85.452
4	2.239	4.072	94.644	2.239	4.072	94.644	5.056	9.192	94.644
5	1.196	2.175	96.819						
6	1.060	1.927	98.746						
7	0.221	0.401	99.147						
8	0.163	0.296	99.444						
9	0.132	0.240	99.683						
10	0.071	0.129	99.812						

^a^Extraction method: principal component analysis.

The first factor (F1) (accounting for 43.604% of the total variance) has a good relationship with C1 (r = 0.896, p < 0.001) (Table 2). F2 (accounting for 23.181% of the total variance) has a good relationship with C2 (r = 0.943, p < 0.001). The correlation between F3 and C3 is poor (r = 0.333); however, there is a good negative correlation between C2 and C3 (r = −0.687, p < 0.001). In addition, C4 has good correlations with F3 (r = 0.566, p < 0.001) and F4 (r = 0.717, p < 0.001). The above analysis shows that F1 controlled the trend of the environmentally sensitive component C1. F2 controlled the trend of the sensitive component C2. There was a significant negative correlation between C2 and C3, which was affected by the same factor, F2. F3 and F4 together influenced the sensitive component C4. Additionally, because the content of the C4 component is too small, i.e., the content of sub-population S4 is less than 2.65%, F3 and F4 are not discussed separately.

**Table 2:** The Pearson correlation coefficient matrix among the four factor scores (F1, F2, F3, and F4) and the contents of the four peaks (C1, C2, C3, and C4) extracted from the curve of the standard deviation versus grain size.

	C1	C2	C3	C4	F1	F2	F3	F4
C1	1	0.269	−0.842^a^	−0.562^a^	0.896^a^	0.310	−0.168	−0.173
C2	0.269	1	−0.687^a^	−0.321	−0.057	0.943^a^	−0.280	−0.073
C3	−0.842^a^	−0.687^a^	1	0.577^a^	−0.592^a^	−0.675^a^	0.333	0.157
C4	−0.562^a^	−0.321	0.577^a^	1	−0.356	−0.125	0.566^a^	0.717^a^
F1	0.896^a^	−0.057	−0.592^a^	−0.356	1	0	0	0
F2	0.310	0.943^a^	−0.675^a^	−0.125	0	1	0	0
F3	−0.168	−0.280	0.333	0.566^a^	0	0	1	0
F4	−0.173	−0.073	0.157	0.717^a^	0	0	0	1

^a^Correlation is significant at the 0.01 level (2-tailed).

## Discussion

Changes in land cover and land use will change the grain size of terrigenous debris^46^, and a change in the lake hydrodynamic intensity will lead to a grain-size redistribution of terrigenous detritus in lake sediments^4^. The organic materials, including TOC and TN, in the lake sediments are mainly controlled by the initial productivity of the lake and by the input of terrigenous organic debris^47,48^. Different sources of organic matter have different C/N ratios. Aquatic phytoplankton are mainly rich in protein and organic nitrogen, so the C/N ratio is low (4 to 10), whereas terrestrial vascular plants are composed mainly of lignin and cellulose, with C/N values of 20–30^49^. The results suggest that the organic matter in the sediments of Bosten Lake was mainly from aquatic plants and therefore reflects the primary productivity in the lake. Starting the early 1960s, the TOC and TN contents significantly decreased in response to the significant decline in water level and increased water salinity caused by enhanced water demands. Since the late 1980s, the water level of the lake has risen, and the lake primary productivity of Bosten Lake has improved. The organic matter in the sediments of Bosten Lake does not reflect changes in land cover in the basin, and thus, it is impossible to explore the influence of changes in vegetation on the particle size of terrigenous debris.

**Figure 6:**
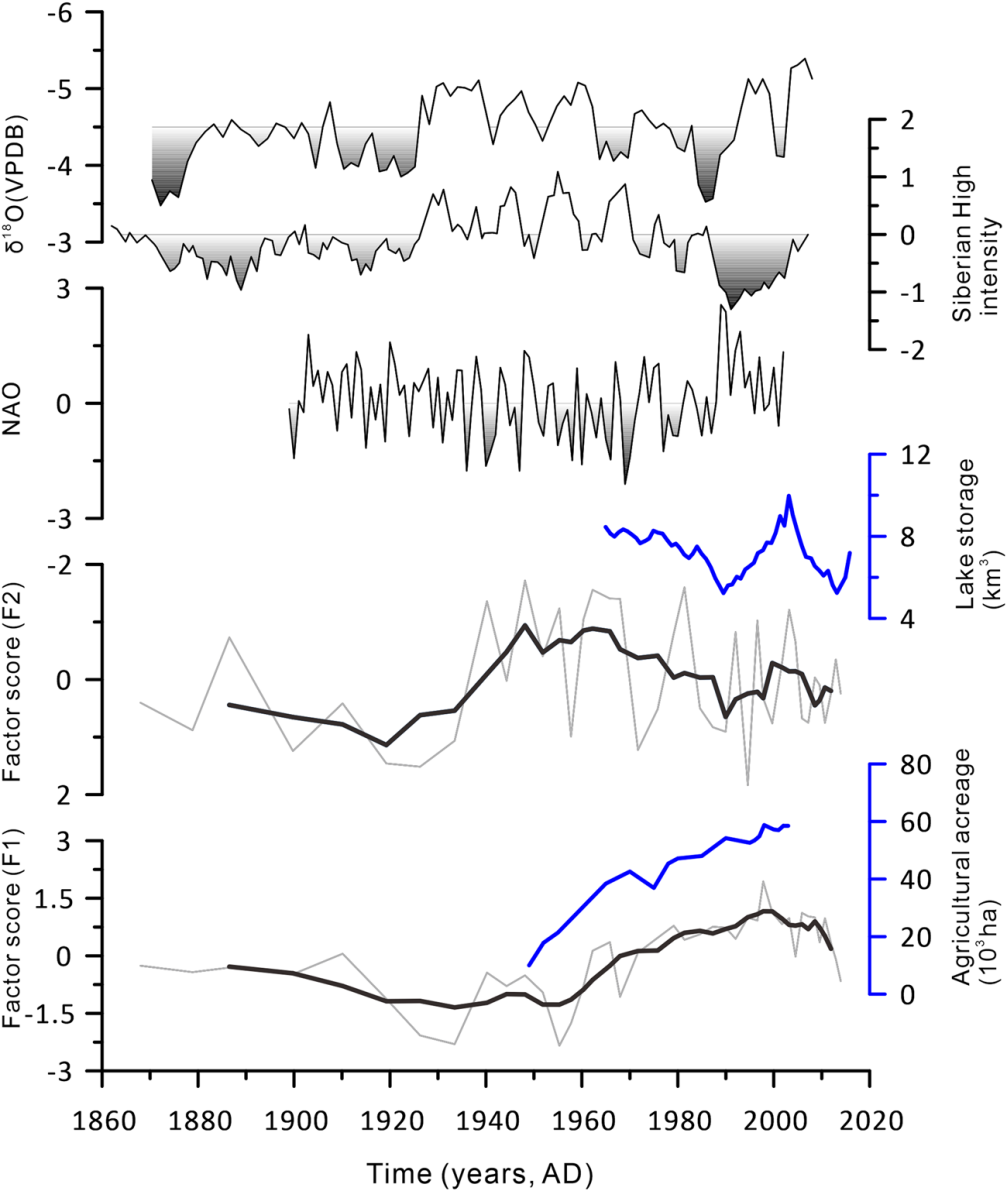
The factor scores of F1 and F2 with five-point running averages compared with regional environmental indicators, including the Siberian High intensity^62^, Indian monsoon index^66^, North Atlantic Oscillation (NAO)^63^, water level of Bosten Lake^15^, and agricultural acreage of the Bosten Lake watershed^67^.

The results of the Pearson correlation analysis show that the factor score F1 is correlated with the content C1 (the 3.31 µm size fraction) (Table 2), indicating that cultivated land, through the alternation of the underlying surface, caused an increase in the atmospheric dust concentration^50–53^. This finding is supported by the fact that the results of the factor analysis show that the five-point running average of the factor score (F1) is consistent with that of the change in the agriculture acreage in the Bosten Lake watershed (Fig. 6). Thus, it can be concluded that increasing human activity caused an increase in aeolian dust deposition in Bosten Lake. Increases in MS values in lake sediments suggest higher land surface erosion in the lake watershed resulting from human activity^54^. From the MS variation (Fig. 3), the land surface erosion caused by human activities in the Bosten Lake watershed has increased significantly since the 1950s. Land surface erosion caused by human activities is superimposed on the natural state, which could also influence the grain size of terrestrial debris. However, the terrigenous clastic materials that entered the lake through surface runoff are affected by the hydrological conditions of Bosten Lake. This can be inferred from the variation in factor F2. The correlation analysis results suggest that the contents C2 (the 11.48 μm size fraction) and C3 (the 69.18 μm size fraction) are mostly sensitive to the influences of factor F2. The five-point running average curve of the factor score F2 is consistent with the trend of lake water storage and therefore reflects environmental aspects of the lake, including lake water level, surface area, and lake inflow runoff.

During the Little Ice Age (1500–1900 AD), the climate was cold and humid in this region^36^. Since 1900 AD, the Bosten Lake region has been warm and dry^36^, and the lake water level has decreased. From the five-point running average curve, we can detect two significant periods with the lowest water storage, that is, 1920–1930 and 1980–1990. The regional drought in the 1920s has been studied using other records from lake sediments and tree rings^55–57^, which show that the climate was dry during this period, indicating that the drought was a regional event. In previous research by Shi *et al*. (2007), a warm-moist transition was found in 1985 AD using instrumental data and data regarding changes in glaciers and lakes over the past 50 years in arid northwestern China^58^.

As highly vulnerable terrestrial ecosystems, the arid regions of Central Asia are heavily influenced by the westerly circulation, the Arctic Ocean airflow and the Indian Monsoon Current, making them sensitive to global changes^59,60^. On the one hand, winter temperatures in this region have a strong relationship with the Siberian High^61^, and an increase in winter temperatures is not conducive to the formation of precipitation. Additionally, weakening of the Siberian High can result the transport of less water vapour from the Arctic Ocean to Xinjiang. During 1920–1930, the Siberian High was weak^62^, and the precipitation in southern Xinjiang was minimal. In addition, it can also be seen that with weakening of the intensity of the Indian monsoon (Fig. 6), the amount of water vapour entering Xinjiang is bound to decrease. In the late 1980s, the North Atlantic Oscillation (NAO) was in the negative phase^63^, as shown in Fig. 6. Water vapour from the Atlantic Ocean, carried by the westerlies, crosses the western mountains into Xinjiang, producing precipitation there. When the NAO is in a negative phase, the westerly wind is relatively weak^64,65^, enabling only a small amount of southwestern warm and humid air to enter Central Asia and resulting in less precipitation there. It can also be seen from Fig. 6 that during this period, the Siberian High was low^62^, and the Indian monsoon was weak^66^, resulting in a decrease in the Indian Ocean water vapour entering Xinjiang. However, since the late 1980s, decreases in water consumption due to drip irrigation, mulching and other agricultural water-saving technologies and enhanced watershed precipitation^67^ have induced an increase in water levels.

The greatest influences on the lake and its catchment since the Little Ice Age have been anthropogenic^68,69^. The characteristics of the formation of and changes in the ecological environment since the Little Ice Age can be used to identify the impact of human activities and to predict future environmental changes. A comparison of the Bosten Lake watershed^15^ and the Aral Sea basin^70^ over the past 50 years reveals that these regions have experienced similar climatic trends. It is interesting that the water storage in these two lakes continued to decline in the 1960s, and the water environment of both lakes was the worst in approximately 1980–1990. A pumping station was constructed in the eastern part of Lake Bosten (Fig. 1) in 1983 AD to adjust the outflow to the Peacock River. Due to prominent contradictions in the allocation of water resources between Central Asian countries and a lack of centralized and effective water resource utilization policies, the inflow of water entering the lake has been consistently low^71^ compared with the increased precipitation in the Aral Sea basin since the late 1980s^70^. It is inferred that human activity has had a profound impact on lake change in arid Central Asia over the past 50 years.

## Conclusions

Using ^137^Cs and ^210^Pb dating of a short, 41-cm lacustrine sediment core from Bosten Lake, Central Asia, the sediment grain-size distribution in response to the changes in the lake environments was studied under climatic and anthropogenic influences, and lake water level changes over the past 150 years were reconstructed. The results are as follows:

1. Environmentally sensitive grain-size components were extracted from the grain-size standard deviation method and factor analysis. The components with grain sizes of 11.48 μm and 69.18 μm were sensitive to variations in the lake water level.
2. The history of lake water level changes over nearly 150 years has been established, thus extending the 50-year instrumental record. There are two significant periods with the lowest water level: 1920–1930 AD and 1980–1990 AD. Weakening of the Siberian High and reduction of the water vapour transported from the Indian Ocean during this period led to a reduction in the local water vapour, which resulted in reduced lake levels in 1920–1930 AD.

## Methods

### Sampling and laboratory analysis

In June 2016, a lake sediment sampler (Uwitec, Austria) was used to extract a short sediment core with a length of 41 cm (41°55.665′N, 86°49.078′E) from a depth of 7.0 m in Bosten Lake (Fig. 1). The sediment core was sliced at 1 cm intervals *in situ*. The freeze-dried sub-samples were analysed for ^210^Pb, ^226^Ra and ^137^Cs by an EG&G Ortec gamma spectrometer (HPGe GWL-120-15), while the activity of ^226^Ra in the lake sediment was evaluated by averaging the activities of ^214^Pb (295 keV and 352 keV) and ^214^Bi (609 keV). The total ^210^Pb was 46.5 keV, and the total ^137^Cs was 662 keV, with standard counting errors of less than 10%^72^.

After treating samples with 1 N HCl, the determination of total organic carbon (TOC) and total nitrogen (TN) were performed using a CE-440 elemental analyser (EAI Company)^73^. Magnetic susceptibility was measured using a Bartington MS2 meter (Bartington Instruments Ltd.), and the detailed procedure is described in reference ^68^. Organic matter and carbonates were removed from the sediment samples with 10–20 ml of 30% H_2_O_2_ and 10 ml of 10% HCl. Then, 10 ml of 0.05 M (NaPO_3_)_6_ was added to the residues, which were then subjected to ultrasonic treatment for 10 min. Grain-size analysis of the lake sediments was carried out using a Malvern Mastersizer 2000 with a Hydro 2000 MU dispersion unit (Malvern, Worcestershire, UK) with a relative error of <1%. The percentages of the related size fractions of a sample were calculated by the Mastersizer 2000^68^.

### Data analysis

The factor analysis model^74,75^ was used to explain the potential factors affecting the grain-size composition of the lacustrine sediments. The data matrix D can be formulated by the equation D[m,r] = C[m,n] × R[n,r], where D represents the grain size data matrix; C represents the factor-loading matrix representing the latent factor composition; and R represents the factor score matrix for factor contributions. In addition, m, n, and r are the number of grain-size fractions, factor, and sample number, respectively. The Pearson correlation method^76,77^ was used to establish the quantitative relationship between the various variables. The curve of standard deviation values versus the grain size of the sediments^68,78,79^ was calculated to reveal the environmentally sensitive grain-size population.
